# Supplementary material for: Cryogenic contrast-enhanced microCT enables nondestructive 3D quantitative histopathology of soft biological tissues
Source: Nat Commun. 2022 Oct 20;13:6207. doi: 10.1038/s41467-022-34048-4 (PMC9584947; doi:10.1038/s41467-022-34048-4)
Supplement: Supplementary file 1 — Supplementary Information [file 41467_2022_34048_MOESM1_ESM.pdf]

Supplementary Figures and Tables

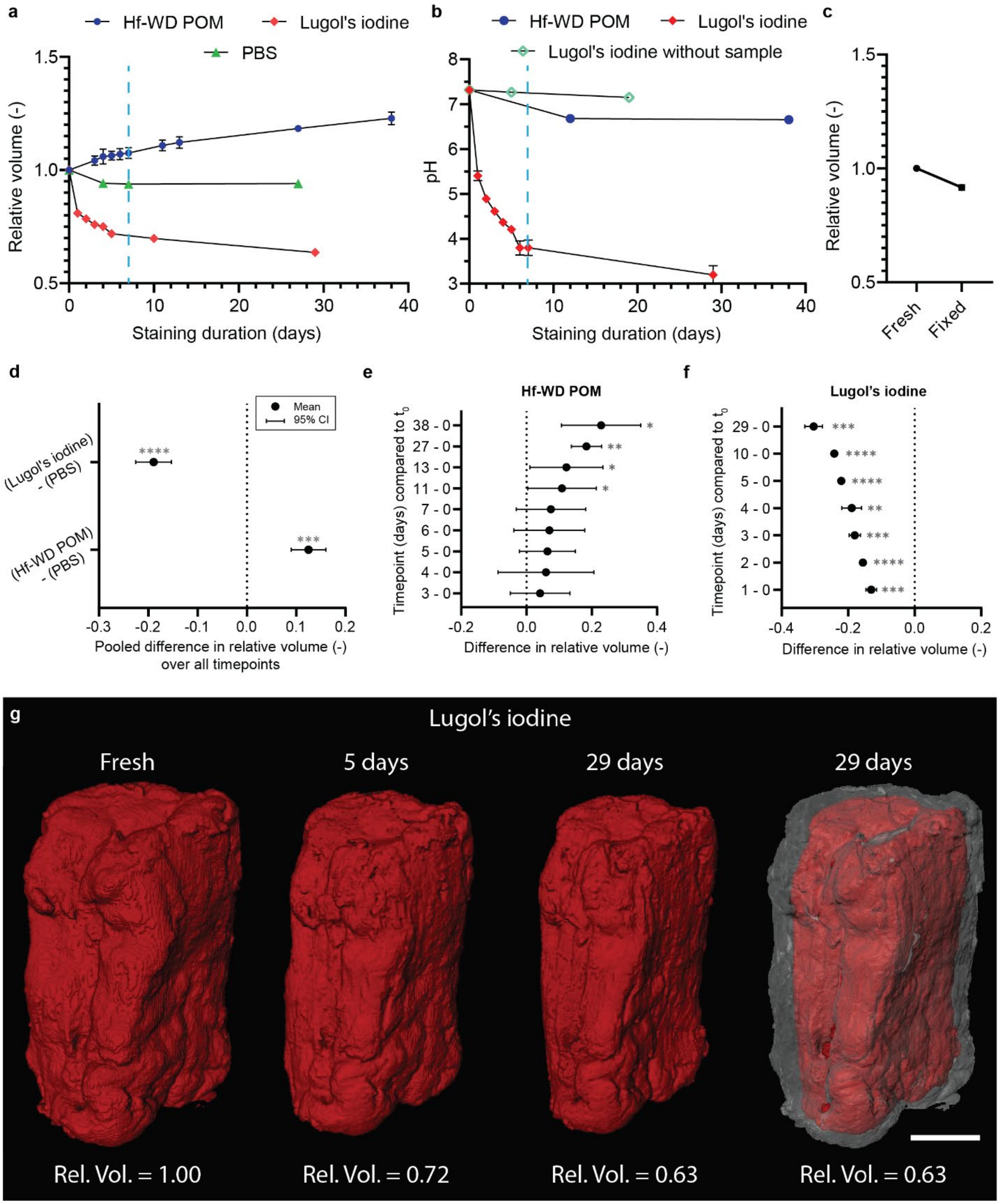

Supplementary Fig. 1| CESA-induced volume changes during staining and the link with the pH of the staining solution. a-c, Volume changes relative to the fresh state (before fixation), measured based on (CE)CT data by imaging bovine muscle samples during the staining process, followed by image segmentation. The data points in the graphs (a-c) represent the mean value (n = 3 for each condition); error bars represent the standard deviation. **a,** The relative volume of the bovine muscle samples as a function of staining time, both for the PBS control group and for the staining with either Lugol's iodine or Hf-WD POM. The staining time used in this study (7 days) is indicated by the blue dashed line. **b,** The pH of the staining solutions as a function of staining time. The staining time used in this study (7 days) is indicated by the blue dashed line. **c**, The relative volume change caused by overnight fixation in PBS buffered 4% formaldehyde solution. **d-f,** Statistical analyses of the effect of CESA (d) and staining time (e-f) on the relative volume changes. In the graphs (d-f), the data points and the bars represent the mean and the 95 % confidence interval, respectively. The pooled difference in relative volume over all the timepoints, both for Hf-WD POM and Lugol's iodine, compared to the PBS control group (n = 3 for each staining condition), was analyzed using a mixed-effects analysis (two-way ANOVA); multiple comparisons were performed using a two-sided Dunnett's test (**d**). The evolution of the difference in relative volume at the different staining time points, both for Hf-WD POM (**e**) and Lugol's iodine (**f**), compared to the initial state, was analyzed using one-way ANOVA and multiple comparisons were carried out using a two-sided Dunnett's test (n = 3 for each staining condition). **g,** Volume renderings of the muscle tissue sample stained with Lugol's iodine at several time points during staining. The relative volume (Rel. Vol.) is also indicated at each time point. The rendering on the right shows an overlay of the volume after 29 days of staining (red) and the fresh volume (white translucent). Scale bar corresponds to 2.5 mm. Significant p-values ($p < 0.05$) have been indicated in the graphs.

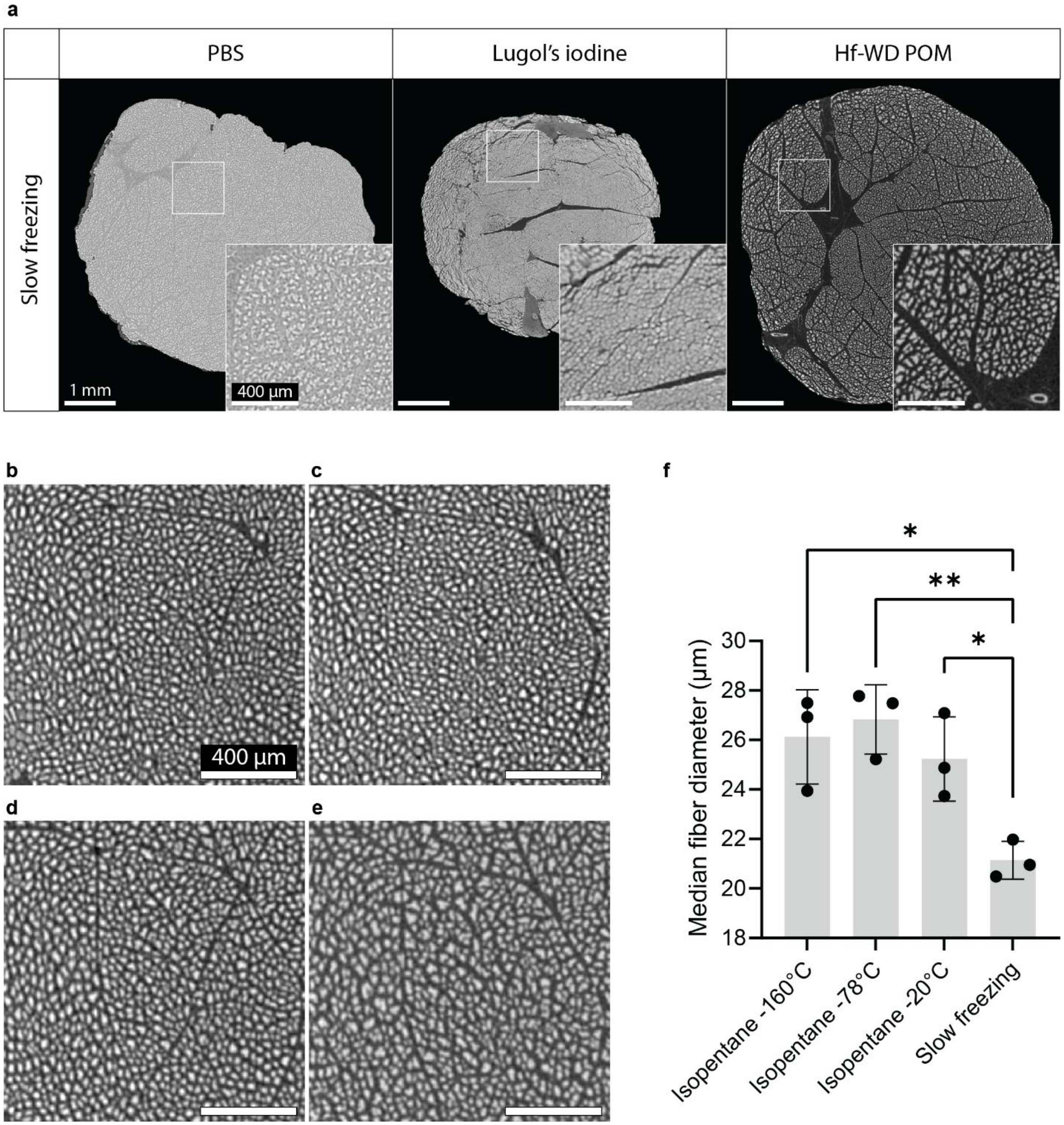

Supplementary Fig. 2|The freezing rate affects the microstructure of bovine muscle tissue, as visualized by cryo-CECT. a, Transverse cryo-(CE)CT images showing the fibrous microstructure after slow freezing in air at -80°C of bovine muscle tissue, without staining (PBS) or after staining with either Lugol's iodine of Hf-WD POM. **b-e,** Transverse cryo-CECT images showing the cross section of the individual muscle fibers, stained with Hf-WD POM, following different freezing methods: freezing by submersion in isopentane at -160°C (**b**), -78°C (**c**) and -20°C (**d**), and slow freezing in air at -80°C (**e**). **f,** Comparison of the median fiber diameter following the different freezing methods. The bars represent the mean, and the error bars indicate the standard deviation; n = 3 for each freezing rate, with > 750 individual fibers measured in each VOI. For comparison of the different freezing rates, one-way analysis of variance with repeated measures, followed by a two-sided Tukey's test, was conducted. Significant p-values ($p < 0.05$) have been indicated in the bar graphs.

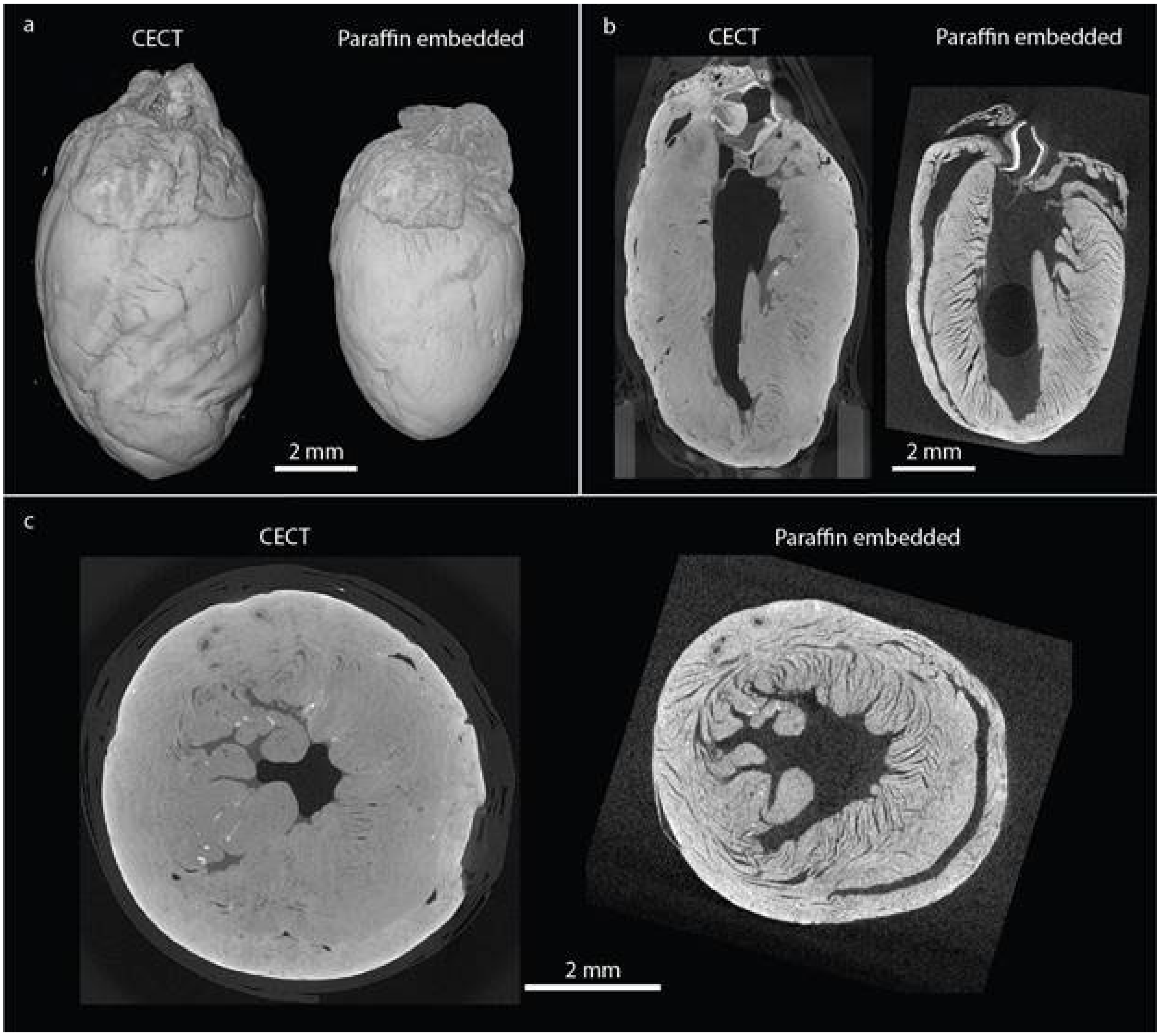

Supplementary Fig. 3| Tissue shrinkage of a murine heart due to classical 2D histological sample preparation. a-c, CECT images of the same heart prior to (CECT; volume = 150 mm³) and after classical 2D histological sample preparation (paraffin embedded; volume = 97 mm³), visualized by 3D renderings (**a**), vertical slices (**b**) and horizontal slices (**c**). Slices have been registered to show the same location for CECT and paraffin embedded.

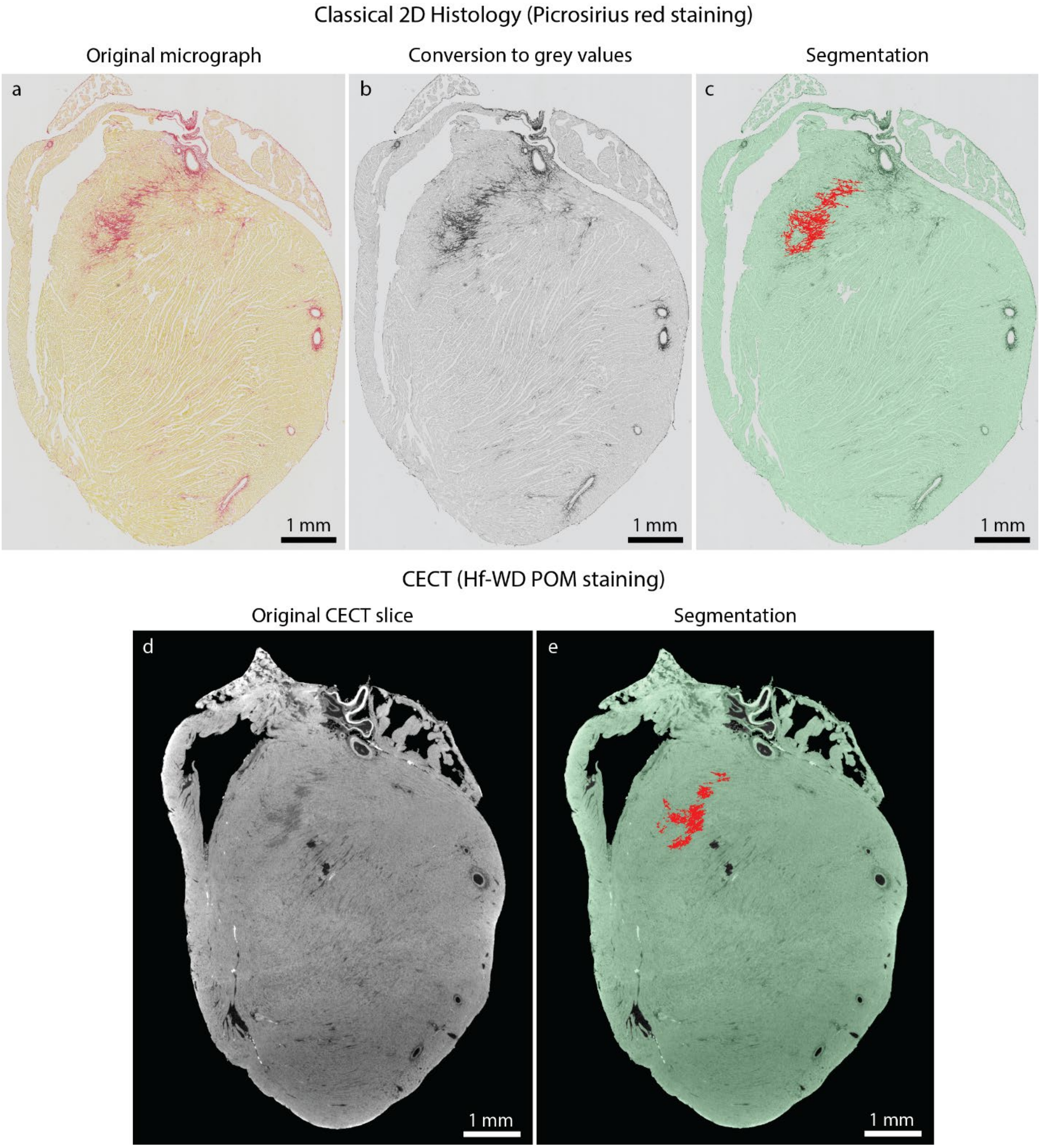

Supplementary Fig. 4 | Quantitative comparison of severe interstitial fibrotic area fraction as measured by classical 2D histology and CECT. a-c, Longitudinal section of a murine heart subjected to TAC, obtained by classical 2D histological sectioning followed by picrosirius red staining (**a**). The section was converted to grey values using the green channel (**b**) to allow segmentation of the entire cardiac tissue (green overlay) and the severe fibrotic region (red) (**c**). **d-e**, Longitudinal CECT image of the same murine heart (**d**), which had been registered to the 2D histological section shown in (a). The entire cardiac tissue (green) and severe fibrotic region (red) was segmented (**e**). Dividing the area of the fibrotic region (red) by the area of the entire cardiac tissue (green) resulted in area fractions of 1.11% and 0.83% for classical 2D histology and CECT, respectively.

Supplementary Table 1| Body weight (BW) before and after TAC/sham surgery, as well as the peak velocity measured by echocardiographic analysis of the mice.

Surgery	Substrain	Sex	BW date of surgery (g)	BW date of sacrifice (g)	Peak velocity (mm/s)
Sham	C57BL6/J	F	20.1	20.4	712
Sham	C57BL6/J	F	19.8	19.8	747
Sham	C57BL6/J	F	18.3	18.6	785
TAC-27G	C57BL6/J	F	21.5	23.0	3855
TAC-27G	C57BL6/J	F	20.7	20.3	4215
TAC-27G	C57BL6/J	F	19.5	20.4	2750
TAC-27G	C57BL6/J	F	21.0	21.1	3150

Supplementary Table 2| Input parameters for the 3D fiber analysis using the modules *Cylinder Correlation* and *Trace Correlation Lines* in the Avizo software.

**Cylinder Correlation**			
Input parameter	***Muscle Hf-WD POM FF (Fig. 2)***	***Muscle Hf-WD POM SF (Fig. 2)***	***Murine hearts (Fig. 5)***
Cylinder length (µm)	200	200	70
Angular sampling (°)	5	5	5
Mask cylinder radius (µm)	22	20	6.4
Outer cylinder radius (µm)	15	13	5.8
Inner cylinder radius (µm)	0	0	0
**Trace Correlation Lines**			
Input parameter	***Muscle Hf-WD POM FF (Fig. 2)***	***Muscle Hf-WD POM SF (Fig. 2)***	***Murine hearts (Fig. 5)***
Minimum seed correlation	68	68	110
Minimum continuation quality	45	45	75
Direction coefficient	0.3	0.3	0.2
Minimum distance (µm)	30	26	11.6
Minimum length (µm)	200	200	100
Search cone length (µm)	200	200	20
Search cone angle (°)	37	37	30
Minimum step size (%)	10	10	10
