# Supplementary material for: Cryogenic contrast-enhanced microCT enables nondestructive 3D quantitative histopathology of soft biological tissues
Source: Nat Commun. 2022 Oct 20;13:6207. doi: 10.1038/s41467-022-34048-4 (PMC9584947; doi:10.1038/s41467-022-34048-4)
Supplement: Supplementary file 2 — Description of Additional Supplementary Files [file 41467_2022_34048_MOESM2_ESM.pdf]

Description of Additional Supplementary Files

Supplementary Movie 1

3D orientation analysis of individual cardiac muscle fibers in an entire murine heart. 3D renderings of the fiber model obtained by the fiber orientation analysis, for a TAC heart and a sham heart. The polar angle of each individual heart muscle fiber is indicated by the color scale. Clipping of the 3D rendering reveals the internal organization and orientation of the fibers.

Supplementary Movie 2

3D localization of severe fibrotic regions in relation to the cardiac muscle fiber orientation. 3D rendering showing the regions of severe interstitial fibrosis (blue regions), overlaid with the fiber orientation map. Clipping of the 3D rendering shows the localization of the severe fibrotic regions in relation to the cardiac muscle fiber orientation. The polar angle of each individual heart muscle fiber is indicated by the color scale.
