# Supplementary material for: Cryogenic contrast-enhanced microCT enables nondestructive 3D quantitative histopathology of soft biological tissues
Source: Nat Commun. 2022 Oct 20;13:6207. doi: 10.1038/s41467-022-34048-4 (PMC9584947; doi:10.1038/s41467-022-34048-4)
Supplement: Supplementary file 5 — Reporting Summary [file 41467_2022_34048_MOESM5_ESM.pdf]

nature portfolio

Corresponding author(s): Greet Kerckhofs

Last updated by author(s): Sep 1, 2022

Reporting Summary

Nature Portfolio wishes to improve the reproducibility of the work that we publish. This form provides structure for consistency and transparency in reporting. For further information on Nature Portfolio policies, see our Editorial Policies and the Editorial Policy Checklist.

Statistics

For all statistical analyses, confirm that the following items are present in the figure legend, table legend, main text, or Methods section.

n/a	Confirmed	
☐	☒	The exact sample size (*n*) for each experimental group/condition, given as a discrete number and unit of measurement
☐	☒	A statement on whether measurements were taken from distinct samples or whether the same sample was measured repeatedly
☐	☒	The statistical test(s) used AND whether they are one- or two-sided<br>*Only common tests should be described solely by name; describe more complex techniques in the Methods section.*
☒	☐	A description of all covariates tested
☐	☒	A description of any assumptions or corrections, such as tests of normality and adjustment for multiple comparisons
☐	☒	A full description of the statistical parameters including central tendency (e.g. means) or other basic estimates (e.g. regression coefficient) AND variation (e.g. standard deviation) or associated estimates of uncertainty (e.g. confidence intervals)
☐	☒	For null hypothesis testing, the test statistic (e.g. *F*, *t*, *r*) with confidence intervals, effect sizes, degrees of freedom and *P* value noted<br>*Give P values as exact values whenever suitable.*
☒	☐	For Bayesian analysis, information on the choice of priors and Markov chain Monte Carlo settings
☒	☐	For hierarchical and complex designs, identification of the appropriate level for tests and full reporting of outcomes
☒	☐	Estimates of effect sizes (e.g. Cohen's *d*, Pearson's *r*), indicating how they were calculated

Our web collection on statistics for biologists contains articles on many of the points above.

Software and code

Policy information about availability of computer code

Data collection	MicroCT data was acquired and reconstructed using phoenix datos|x CT software (commercial; GE Measurement and Control Solutions, Germany; v2.7.1). An in-house developed MATLAB (version R2019b) script was used to convert the reconstructed 16-bit slices (.tiff) to 8-bit slices (.bmp), while simultaneously windowing the histogram range to the dynamic range of the dataset (custom; https://github.com/contrast-team/histogram-windowing). Scans of classical 2D histological sections were acquired using the Leica SCN400 Client software (commercial; v1.5.1).
Data analysis	MicroCT data was analyzed using Avizo software (commercial; version Avizo 3D 2022.1). DataViewer software (Bruker MicroCT, Kontich, Belgium; version 1.5.6.2) was applied to manually align the microCT slices to the histological sections. Statistical analysis was performed using GraphPad Prism 9 software (commercial; v9.0.0).

For manuscripts utilizing custom algorithms or software that are central to the research but not yet described in published literature, software must be made available to editors and reviewers. We strongly encourage code deposition in a community repository (e.g. GitHub). See the Nature Portfolio guidelines for submitting code & software for further information.

Data

Policy information about availability of data

All manuscripts must include a data availability statement. This statement should provide the following information, where applicable:

- Accession codes, unique identifiers, or web links for publicly available datasets
- A description of any restrictions on data availability
- For clinical datasets or third party data, please ensure that the statement adheres to our policy

The microCT datasets generated during and/or analysed during the current study are not publicly available due to their considerable size. However, they are

available upon request to the corresponding author.

Field-specific reporting

Please select the one below that is the best fit for your research. If you are not sure, read the appropriate sections before making your selection.

[x] Life sciences   [ ] Behavioural & social sciences   [ ] Ecological, evolutionary & environmental sciences

For a reference copy of the document with all sections, see nature.com/documents/nr-reporting-summary-flat.pdf

Life sciences study design

All studies must disclose on these points even when the disclosure is negative.

Sample size	No statistical method was used to predetermine the sample size for each study. Since this study introduced a novel imaging technique, we primarily aimed to demonstrate the potential and repeatability of the technique on different types of tissues/organs, both healthy and pathological, instead of proving pathological evidence. Hence, the sample size in our study was limited to n=3 or 4 for each condition, which was deemed to be sufficient for the proof-of-concept of this technique as a 3D histopathological tool.
Data exclusions	No data were excluded.
Replication	Experiments on bovine muscle tissue have not been replicated in their entirety. However, certain conditions such as Hf-WD POM staining + isopentane -78°C freezing have been succesfully replicated in the following separate experiments: (i) Effect of CESA and freezing rate, (ii) Effect of long-term storage at -20°C, (iii) Effect of long-term storage at -78°C. In each bovine muscle tissue experiment, a replicate number of n = 3 was used. The relatively low variability on median fiber diameter demonstrates the reproducibility.<br>The murine heart experiments have been succesfully replicated in follow-up studies, both for healthy and pathological hearts (not published yet). Fiber diameter, fiber orientation and visualization of fibrotic regions were in line with the results shown in this study.
Randomization	For the bovine muscle experiments, all samples within one experiment originated from the same animal (technical replicates), because this study aimed to demonstrate the technical repeatibility of this technique. For each experiment, samples from a different animal were used to avoid potential tissue degradation over time. The samples (small muscle strips) within each experiment were randomly assigned to the experimental groups.<br>Mice were randomly assigned to either the sham group or the transverse aortic constriction-operated group.
Blinding	For the bovine muscle tissue and the murine hearts experiments, blinding was not relevant since the 3D data analysis was performed automatically in the software, using the same image processing and analysis steps for all groups, thus omiting potential user bias.

Reporting for specific materials, systems and methods

We require information from authors about some types of materials, experimental systems and methods used in many studies. Here, indicate whether each material, system or method listed is relevant to your study. If you are not sure if a list item applies to your research, read the appropriate section before selecting a response.

Materials & experimental systems

n/a	Involved in the study	
[x]	[ ]	Antibodies
[x]	[ ]	Eukaryotic cell lines
[x]	[ ]	Palaeontology and archaeology
[ ]	[x]	Animals and other organisms
[x]	[ ]	Human research participants
[x]	[ ]	Clinical data
[x]	[ ]	Dual use research of concern

Methods

n/a	Involved in the study	
[x]	[ ]	ChIP-seq
[x]	[ ]	Flow cytometry
[x]	[ ]	MRI-based neuroimaging

Animals and other organisms

Policy information about studies involving animals; ARRIVE guidelines recommended for reporting animal research

Laboratory animals	Bovine muscle samples (Hereford cows; 18-24 months) were supplied by a local farm (Jos Theys Boerderij).<br>The Achilles bone-tendon insertion was harvested from a Landrace female pig (6 months; weighing 40kg), which was used within the frame of an ongoing experiment from our collaborators.<br>Murine hearts were harvested from wild type C57BL/6J, female mice (11-12 weeks of age).
Wild animals	The study did not involve wild animals.
Field-collected samples	The study did not involve samples collected from the field.

Ethics oversight	Animal handling and experimental procedures were approved by the local authorities (Comité d'éthique facultaire pour l'expérimentation animale, 2021/UCL/MD/009, UCLouvain, Belgium) and performed in accordance with the Guide for the Care and Use of Laboratory Animals, published by the US National Institutes of Health (NIH Publication, revised 2011). All animals were housed with a 12 h/12 h light/dark cycle, with the dark cycle occurring from 6.00 p.m. to 6.00 a.m. Mice were observed daily with free access to water and standard chow.<br><br>Since the bovine muscle samples were supplied by a local farm (cows were sacrificed for food purposes) and the porcine Achilles insertion sample was a recuperation of experimental material, no ethical approval was required for these samples.

Note that full information on the approval of the study protocol must also be provided in the manuscript.
